# Motile Cilia: Innovation and Insight From Ciliate Model Organisms

**DOI:** 10.3389/fcell.2019.00265

**Published:** 2019-11-01

**Authors:** Brian A. Bayless, Francesca M. Navarro, Mark Winey

**Affiliations:** ^1^Department of Biology, Santa Clara University, Santa Clara, CA, United States; ^2^Department of Molecular and Cellular Biology, University of California, Davis, Davis, CA, United States

**Keywords:** motile cilia, basal body, ciliate, doublet microtubules, ciliary array

## Abstract

Ciliates are a powerful model organism for the study of basal bodies and motile cilia. These single-celled protists contain hundreds of cilia organized in an array making them an ideal system for both light and electron microscopy studies. Isolation and subsequent proteomic analysis of both cilia and basal bodies have been carried out to great success in ciliates. These studies reveal that ciliates share remarkable protein conservation with metazoans and have identified a number of essential basal body/ciliary proteins. Ciliates also boast a genetic and molecular toolbox that allows for facile manipulation of ciliary genes. Reverse genetics studies in ciliates have expanded our understanding of how cilia are positioned within an array, assembled, stabilized, and function at a molecular level. The advantages of cilia number coupled with a robust genetic and molecular toolbox have established ciliates as an ideal system for motile cilia and basal body research and prove a promising system for future research.

## Introduction

Ciliates are single-celled protozoans grouped under the phylum Ciliophora (Cavalier-Smith, 1993). Species of ciliates are morphologically diverse but, as their name suggests, ciliates are united in that they contain many motile cilia. *Paramecium tetaurelia/caudatum* and *Tetrahymena thermophila* are the two ciliate species that have had the biggest impact on our understanding of both motile cilia and their major nucleating/anchoring structure, the basal body.

**FIGURE 1:**
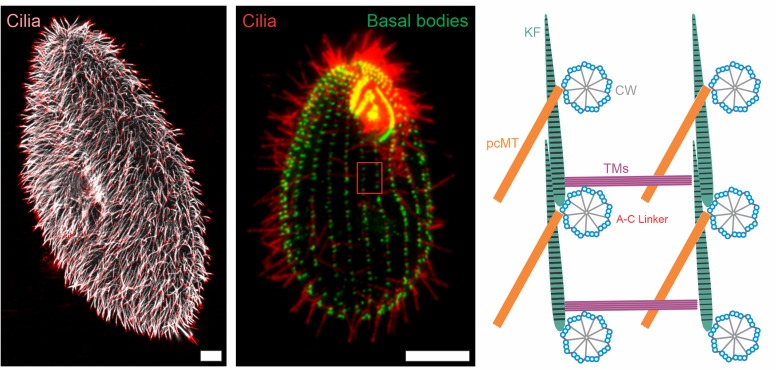
Immunofluorescence images and schematic representation of ciliate cilia and accessory structures. Immunofluorescence images of *Paramecium tetaurelia* **(left)** and *Tetrahymena thermophila* **(middle)** cells. The *Paramecium tetaurelia* cell **(left)** is stained for cilia (α-GT335, white; α-TAP952, red) and the *Tetrahymena thermophila* cell **(middle)** is stained for basal bodies (α-centrin, green) and cilia (α-GT335, red). Scale bar = 10 μm. The red box in the *Tetrahymena* image **(middle)** is represented schematically in the **right** image showing a top down view of basal body organization with associated accessory structures. CW, cartwheel; pcMT, post ciliary microtubules; TM, transverse microtubules; KF, Kinetodesmal Fiber.

Both *Tetrahymena* and *Paramecium* are genetically tractable systems. They have completely sequenced genomes (Aury et al., 2006; Eisen et al., 2006) and their genetics, though unconventional, lend themselves well to both forward and reverse genetic approaches, including genomic knockouts, knock-ins, and gene fusions (Bruns and Cassidy-Hanley, 2000; Hai et al., 2000; Kung et al., 2000; Yu and Gorovsky, 2000; Shang et al., 2002b; Beisson et al., 2010). Importantly, ciliate cilia are structurally and molecularly conserved with higher eukaryotes (Carvalho-Santos et al., 2011). Their cilia are arranged in an array that is comparable to human multi-ciliated cells, and their cilia beat in a biphasic whip-like motion that is consistent with their human counterparts (Wood et al., 2007; Funfak et al., 2015). Perhaps the most important advantage that ciliates have for cilia and basal body research is their large quantity of cilia per cell. A typical vertebrate multi-ciliated epithelial cell contains 200–300 cilia per cell, however, *Tetrahymena* and *Paramecium* cells contain upward of 750 and 4000 cilia per cell, respectively (Figure 1 and Supplementary Table S1; Pearson and Winey, 2009; Tilley et al., 2015). The large quantity of cilia has established ciliates as an ideal system for both light and electron microscopy as well as proteomics. In the following sections of this review we will specifically focus on the joint contributions of *Tetrahymena* and *Paramecium* to our understanding of basal body and motile cilia composition, assembly, structure, and function.

## Basal Bodies

Basal bodies nucleate and anchor motile cilia in the cell, and as such, they are essential for the function of motile cilia. The basal bodies of ciliates provide a number of advantages for researchers. Ciliate basal bodies are strikingly similar to those of other eukaryotes in both structure and molecular composition (Carvalho-Santos et al., 2011). They are assembled in a stepwise process that is analogous to that of centrioles; however, ciliate basal bodies never function as centrioles (Dippell, 1968; Allen, 1969). This is experimentally advantageous because basal body defects are never conflated with mitotic defects. Additionally, like the multi-ciliated cells found in higher eukaryotes such as humans and *Xenopus laevis*, ciliate basal bodies are positioned and oriented into an array via a comprehensive network of accessory structures (Figure 1 and Supplementary Table S1) (Bayless et al., 2015; Tassin et al., 2015; Zhang and Mitchell, 2015; Vertii et al., 2016). Methods have also been developed to isolate and purify basal bodies, leaving researchers with pure fractions to perform biochemical and proteomic studies (Argetsinger, 1965; Tiedtke, 1985).

### Identification of Basal Body Components

A proteome of *Tetrahymena* basal bodies was completed in 2007 (Kilburn et al., 2007). The *Tetrahymena* proteome displays a significant amount of overlap with two other basal body proteomes from human centrioles and *Chlamydomonas* basal bodies, a finding that highlights the high molecular conservation between phylogenetically distant species (Andersen et al., 2003; Keller et al., 2005). Importantly, the *Tetrahymena* proteome advanced the field by localizing identified proteins through GFP tagging and immuno-EM mapping (Kilburn et al., 2007). This localization data has served as an important first step in the functional characterization of many components, including the core basal body components Bld10, Poc1, and Sas6 (Culver et al., 2009; Pearson et al., 2009; Bayless et al., 2012; Meehl et al., 2016).

### Basal Body Assembly and Maintenance

Basal body assembly begins with the formation of a radially symmetric cartwheel followed by the addition and elongation of triplet microtubules to the ends of each cartwheel spoke. Adjacent triplet microtubules are connected by A-C linkers then the entire basal body is capped at its distal end in a region called the transition zone, where the triplet microtubule of the basal body give way to the doublet microtubules of the ciliary axoneme (Figure 1; Carvalho-Santos et al., 2010). The first structural and temporal dissection of early basal body assembly was completed in ciliates in the late 1960’s (Dippell, 1968; Allen, 1969). These comprehensive cytological studies used electron microscopy to identify the early stages of basal body assembly, including the temporal ordering of assembly of the cartwheel and the microtubule walls (Dippell, 1968; Allen, 1969). In addition, these studies clearly define the major structural domains of the basal body like the cartwheel, A-C linkers, and the transition zone. Several of these structures are well defined in recent electron tomography studies (Höög et al., 2012; Li et al., 2012; Meehl et al., 2016; Greenan et al., 2018).

At the heart of basal body structure is the triplet microtubules. Triplet microtubule assembly and stability rely on a number of non-canonical tubulins, and ciliates have been impactful in their study and classification. The essential role of γ-tubulin in the earliest stages of basal body microtubule nucleation was first described in ciliates (Ruiz et al., 1999; Shang et al., 2002a). δ-tubulin has been shown in *Paramecium* to be necessary for the assembly of the basal body C-tubule (Garreau de Loubresse et al., 2001). Rarer tubulin isoforms ε- and ζ-tubulin are also found in ciliates and are necessary for basal body duplication and stabilization (Dupuis-Williams et al., 2002; Ruiz et al., 2004; Ross et al., 2013). *Tetrahymena* has also been used to study how ciliary beating affects the distribution of proteins and tubulin post-translational modifications at the triplet microtubules (Bayless et al., 2016). From this work both tubulin glutamylation and the triplet microtubule localizing protein Fop1 were shown to associate asymmetrically to the region of the basal body that experiences the most compressive force from ciliary beating (Bayless et al., 2016).

In many eukaryotic systems, basal body assembly is initiated by PLK4 (Habedanck et al., 2005). Ciliates have no clear PLK4 homolog, though they do contain many other key proteins involved in early assembly (Carvalho-Santos et al., 2010). Functional studies on the major constituent of the cartwheel, Sas-6, revealed that a fully formed cartwheel is necessary for basal body assembly (Culver et al., 2009). Attachment of the cartwheel to the triplet microtubules is facilitated by Bld10, a protein first identified and characterized in *Chlamydomonas*, though work in ciliates has established that Bld10 is essential for assembly and stability of basal bodies (Matsuura et al., 2004; Hiraki et al., 2007; Jerka-Dziadosz et al., 2010; Bayless et al., 2012). It is not only the link between cartwheels and the triplet microtubules that is necessary for stabilizing the basal body, the A-C linker between adjacent triplet microtubules is necessary as well. *Tetrahymena* Poc1 is necessary for establishing the A-C linker between adjacent triplet microtubules (Meehl et al., 2016). When Poc1 is lost from *Tetrahymena* cells, basal bodies fall apart under conditions where cilia-generated force is increased, highlighting the role of structural connections in maintaining the overall stability of the basal body (Pearson et al., 2009; Meehl et al., 2016).

Work in ciliates has established that Centrin 2 is necessary for basal body elongation and establishment of the transition zone, while Centrin 3 is necessary for anchoring the basal body in place (Guerra et al., 2003; Stemm-Wolf et al., 2005; Vonderfecht et al., 2011, 2012; Aubusson-Fleury et al., 2012; Jerka-Dziadosz et al., 2013). Taken together, these works represent the large impact ciliates have had on our understanding of basal body assembly and stability.

### Basal Body Placement in an Array

The complex organization of basal body arrays have been well studied in ciliates. In both *Paramecium* and *Tetrahymena*, basal bodies are arranged into rows, called kinety, that extend along the anterior-posterior axis of the cell (Figure 1; Lynn, 1981; Iftode and Fleury-Aubusson, 2003). The positioning of basal bodies along a kinetid is complex and utilizes the coordination of at least three accessory structures: transverse microtubules, post-ciliary microtubules, and the striated rootlet (kinetodesmal fiber). The two microtubule based structures appear to be specific to ciliates with the transverse microtubules reaching across kinety and the post-ciliary microtubules reaching behind the basal body within a kinety (Figure 1; Lynn, 1981; Iftode and Fleury-Aubusson, 2003). The striated rootlet forges an attachment from the basal body to the plasma membrane and anchors it into place. Striated rootlets are found in most systems that contain multi-ciliated cells, thus highlighting the importance of this cortical connection (Steinman, 1968; Anderson, 1972; Sandoz et al., 1988). More recent work utilized automated image analysis to reveal the three dimensional arrangement of the *Tetrahymena* ciliary array (Galati et al., 2016). In a landmark study that tested the theory of structural inheritance, Janine Beisson and Tracy Sonneborn generated inverted kinety of basal bodies in *Paramecium* and found that all accessory structures built subsequently became inverted to match the inverted positioning of basal bodies (Beisson and Sonneborn, 1965). This experiment demonstrated that not all information is encoded by DNA, but some is, in fact, epigenetic. A result that was later replicated in *Tetrahymena* (Ng and Frankel, 1977). Furthermore, the kinetodesmal fiber alters its length in response to altered cilia-generated forces, suggesting that structural inheritance is tunable and able to adapt to environmental cues (Galati et al., 2016).

The molecular composition of basal body accessory structures is not well-defined but ciliates have contributed greatly to what we do know. The *Tetrahymena* striated fiber is made up, at least in part, by the DisAp protein (Galati et al., 2014). Orientation of basal bodies is disrupted by loss of *Paramecium* proteins Meckelin and Centrin 3, while ODF-1 and VFL3 are necessary for the docking of *Paramecium* basal bodies to the cell cortex (Jerka-Dziadosz et al., 2013; Picariello et al., 2014; Bengueddach et al., 2017). These studies highlight the power of ciliate reverse genetics approaches. Recent work has also made connections between the tubulin post-translational modification glycylation and attachment of *Tetrahymena* basal bodies at the cell cortex (Junker et al., 2019). The assembly, maintenance, and precise positioning of basal bodies remains an essential foundation for the building of a functional cilium and ciliates have, and will remain, at the forefront of basal body research.

## Motile Cilia

Motile cilia and flagella function to move extra-cellular fluid. Structurally, the ciliate motile cilium is conventional consisting of nine set of doublet microtubules arranged radially around a central pair of single microtubules. These cilia beat in a biphasic, whip-like pattern that is consistent with the motile cilia of human cells (Supplementary Table S1; Tuxhorn et al., 1998; Bayless et al., 2015; Funfak et al., 2015). Experimentally, genetic techniques such as protein tagging allow for easy visualization of cilia and a simple calcium shock is sufficient to shear cilia from the cell allowing for easy isolation for biochemical assays (Rosenbaum and Carlson, 1969; Adoutte et al., 1980). As described below, research using ciliates has done much to illuminate the molecular composition, structure, assembly, and function of motile cilia.

### Identification of Motile Cilia Components

In many of the same ways that ciliates have been impactful for identifying the molecular components of basal bodies, ciliates have also been used successfully in the identification of motile cilia molecular components. The significant overlap between the vertebrate and protist cilia proteome was made clear in a mass spectrometry screen using *Tetrahymena* cilia dubbed the “ciliome”(Smith et al., 2005). Isolation and purification of the *Paramecium* ciliary membrane also yielded a proteome of the ciliary membrane, the first of its kind (Yano et al., 2013). Outside of proteomics, whole genome expression profiling has been used to identify the genes that are upregulated during ciliogenesis, which offers a strong starting point for novel discovery and characterization of ciliogenesis proteins (Arnaiz et al., 2010).

### Motile Cilia Structure

As with basal bodies, electron microscopy has been employed to investigate the structural makeup of ciliate ciliary axonemes. Detailed cytological analysis of the transition zone between basal bodies and ciliary axonemes was carried out in both *Paramecium* and *Tetrahymena* (Dippell, 1968; Allen, 1969; Hufnagel, 1969; Dute and Kung, 1978). At the distal end of the axoneme, the termination of the central pair of microtubules and the ciliary tip were first described in detail in *Tetrahymena* axonemes (Sale and Satir, 1977). *Tetrahymena* axonemes were also used in fine structural work detailing the radial spoke and axonemal dynein complexes that are necessary for ciliary movement (Goodenough and Heuser, 1985).

**FIGURE 2:**
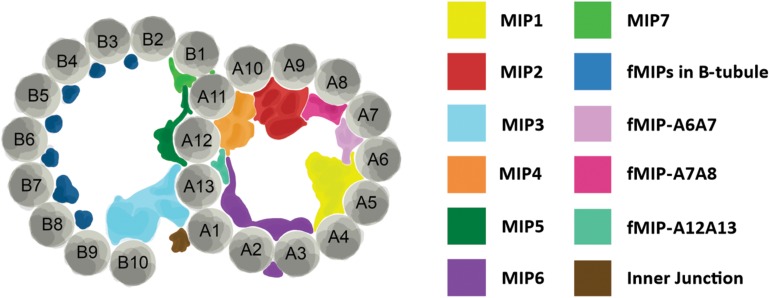
Structure of axoneme doublet microtubules. Cross-section of ciliary axoneme doublet microtubules. A- and B-tubules are shown. Tubulin protofilaments are colored gray and numbered. Microtubule inner proteins (MIPs) and filamentous MIPs (fMIPs) are distinguished by color as denoted in the table to the right. Adapted from Ichikawa and Bui (2018) and Stoddard et al. (2018).

Perhaps the biggest impact that ciliates have made in the area of cilia structure has come from the use of single particle cryo-electron microscopy (cryo-EM) and cryo-electron tomography (cryo-ET) to investigate the structure of the ciliary doublet microtubules. Doublet microtubules are exceptionally stable making them an ideal substrate for cryo-EM and cryo-ET. Early studies used sea urchin sperm flagella for cryo-EM/ET, more recently *Chlamydomonas* flagella and *Tetrahymena* axonemes have become the model systems of choice (Pigino et al., 2012). Currently, single particle cryo-EM of *Tetrahymena* axonemes has achieved the best resolution of doublet microtubules to date at below a nanometer resolution (Ichikawa et al., 2017). There are two areas where the cryo-EM and cryo-ET structural work from *Tetrahymena* axonemes has been influential: our understanding of the machinery outside of the doublet microtubules and our understanding of the interior of the doublet microtubules. Radial spoke proteins and dynein arms make motile cilia move. The structure of these large complexes has been elucidated by structural work in *Chlamydomonas* but also with the help of *Tetrahymena* (Pigino et al., 2011). The lumen of the doublet microtubules has also been shown to have complex networks of proteins in them termed microtubule inner proteins or MIPs (Figure 2; Ichikawa et al., 2017; Ichikawa and Bui, 2018; Stoddard et al., 2018). These MIPs are thought to stabilize the doublet microtubule to allow for the repeated bending without break. The structural studies of doublet microtubules in ciliates have shown us just how complex the axoneme is, highlighting the importance of a precise assembly process.

### Motile Cilia Assembly

Motile cilia assembly occurs through a concerted process called intraflagellar transport (IFT; Nakayama and Katoh, 2018). The advantage that ciliates have for studying ciliary assembly is two-fold. First, ciliates have a large number of cilia. Second, cilia assembly can be induced and followed in real time (Jiang et al., 2015). The first step in assembly of a cilium is to establish a ciliary cap. In *Tetrahymena* this is a multistep process that involves the confluence of three diverse structures (Seixas et al., 2017). After establishment of a ciliary cap, IFT is utilized to assemble the axoneme. In *Paramecium*, IFT46 is necessary for the trafficking of other IFT components between the cytoplasm and the ciliary axoneme, thus making it an upstream regulator of IFT (Shi et al., 2018). In *Chlamydomonas* and *Paramecium* axonemes, failure of IFT to traffic axonemal dynein into the cilium results in short cilia that are non-motile (Fassad et al., 2018). Furthermore, IFT trains have been shown to queue up at the base of *Chlamydomonas* and *Tetrahymena* axonemes while waiting their turn during assembly (Wingfield et al., 2017). Interestingly, the *Tetrahymena* LF4/MOK kinase family member, LF4a, regulates ciliary length by limiting the rate of IFT (Jiang et al., 2019). Besides IFT related mechanisms, ciliates have highlighted many other proteins necessary for assembly of the ciliary axoneme. *Tetrahymena* Dyf-1 is necessary for ciliogenesis as is PHLP2, both of which help with the assembly of axoneme microtubules (Dave et al., 2009; Bregier et al., 2013). The axoneme microtubules also display careful coordination of their length control. In *Tetrahymena* axonemes, FAP256/CEP104 promotes A-tubule elongations while CHE-12/Crescerin and ARMC9 specifically regulate the length of the B-tubule (Louka et al., 2018). Overall, work from ciliates has helped us understand the complexities of ciliary assembly, in particular the nuances of IFT.

### Motile Cilia Function

Ciliary beating is a complex process that requires many molecular interactions. During beating, the ciliary axoneme must maintain a regular waveform all while remaining in concert with its neighboring cilia (Wan, 2018). One of the most fundamental questions about motile cilia was answered with the help of ciliates: How do cilia move? In the 1960’s Ian Gibbons identified and purified axonemal dynein from *Tetrahymena* axonemes (Gibbons, 1963; Gibbons and Rowe, 1965). This was the first microtubule molecular motor identified and its discovery represents a major milestone in cell biology. Follow-up studies using both *Paramecium* and *Tetrahymena* cilia identified how the sliding force between doublet microtubules is translated into the bending force that is seen during beating and the requirement of calcium and calcium signaling for propagation of ciliary beating (Bancroft, 1906; Satir, 1965, 1968; Mogami and Takahashi, 1983; Funfak et al., 2015; Yano et al., 2015). These works represent major advances in our understanding of how cilia beating is facilitated.

At the molecular level, the ease of reverse genetic approaches in ciliates allows for directed investigation of ciliary proteins. The conserved protofilament ribbon protein, Rib72, is not only a MIP, but it is necessary for the localization of a majority of MIPs in the A-tubule of *Tetrahymena* ciliary axonemes (Stoddard et al., 2018). When A-tubule MIPs are lost from axonemes, the structural integrity of the axoneme is compromised resulting in cilia that bend abnormally as they move through the typically rigid power stroke (Stoddard et al., 2018). As a result, their waveform and coordination with neighboring cilia is disrupted suggesting that MIPs play an important role in the structural support for the ciliary axoneme (Figure 2). *Tetrahymena* have also been used to functionally characterize ciliary components of radial spokes and the dynein arms, and both are necessary for proper ciliary beating (Urbanska et al., 2015, 2018).

A final and important area of research that ciliates have contributed greatly to is the study of tubulin post-translational modifications. Acetylation of tubulin is a common post-translational modifications found in stable populations of microtubules. The acetyltransferase MEC-17 is responsible for K40 tubulin acetylation and was first identified in *Tetrahymena* (Akella et al., 2010). Identification and characterization of the TTLL family of tubulin modifying enzymes has also been carried out in *Tetrahymena* (Janke et al., 2005). From these studies we find that tubulin glutamylation and glycylation affect the stability and waveform of ciliary beating in *Tetrahymena* axonemes (Wloga et al., 2008, 2009, 2010; Suryavanshi et al., 2010; Junker et al., 2019). Overall, ciliates have been utilized to demonstrate how motile cilia beat and the molecular players that affect ciliary structure and function.

## Perspectives and Future Outlook

The strengths of ciliate research on cilia lies in their favorable genetics and the abundance of cilia in each cell. The simplicity of basal bodies and cilia isolation, combined with the ease of functional characterization of specific ciliary components, ensures that ciliates will be a viable option for basal body and motile cilia research. The resolution achieved by single particle cryo-EM and cryo-ET of *Tetrahymena* axonemes is exciting and provides a promising platform for future structural work. Ciliates are also poised to play a large part in understanding the biomechanics of ciliary beating. High speed imaging of ciliary beating is possible in ciliates and its use, coupled with genetic study of specific proteins, will be important for understanding how the whip-like ciliary beat stroke is performed and maintained (Funfak et al., 2015; Stoddard et al., 2018). Cilia disassembly is not well understood and the ease of inducing deciliation in ciliates could prove powerful for future research. Further, ciliates are positioned well for the study of human ciliary disorders. Genetic variants of Primary Ciliary Dyskinesia (PCD) have been modeled in *Paramecium* offering unparalleled insight into the pathology of this devastating disorder (Fassad et al., 2018). Given advances that have already been made, and blossoming avenues ripe for study, there is reason to believe that the future of ciliate research is bright and the best is yet to come.

## Author Contributions

BB and MW conceived and wrote the manuscript. FN generated the figures and legends.

## Conflict of Interest

The authors declare that the research was conducted in the absence of any commercial or financial relationships that could be construed as a potential conflict of interest.

## Supplementary Material

TABLE S1 A comparison of common model organisms used in motile cilia research.
