# Supplementary figures and images for: Motile Cilia: Innovation and Insight From Ciliate Model Organisms
Source: Front Cell Dev Biol. 2019 Nov 1;7:265. doi: 10.3389/fcell.2019.00265 (PMC6838636; doi:10.3389/fcell.2019.00265)

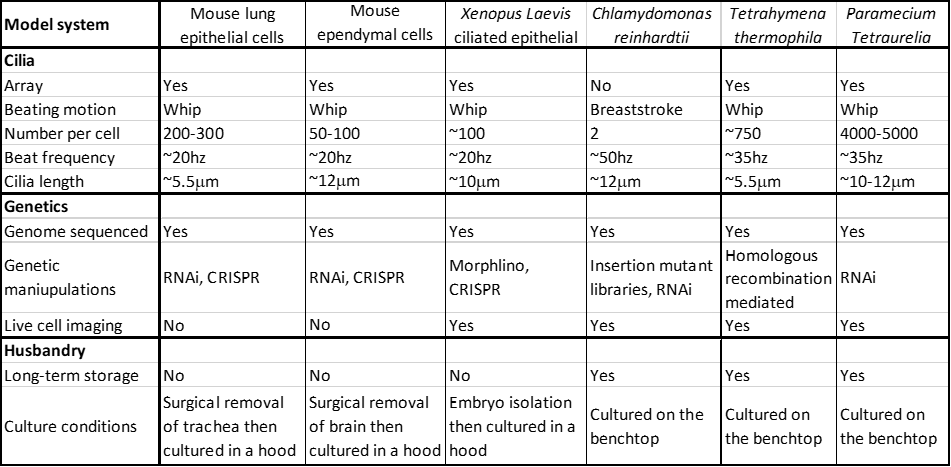

Supplement: TABLE S1 — A comparison of common model organisms used in motile cilia research. [file Image_1.TIF]
